# Virtual Sensor of Surface Electromyography in a New Extensive Fault-Tolerant Classification System

**DOI:** 10.3390/s18051388

**Published:** 2018-05-01

**Authors:** Karina de O. A. de Moura, Alexandre Balbinot

**Affiliations:** Electrical Engineering, Instrumentation Laboratory, Federal University of Rio Grande do Sul (UFRGS), Avenue Osvaldo Aranha 103, Porto Alegre, RS 90035-190, Brazil; alexandre.balbinot@ufrgs.br

**Keywords:** biomedical signal modelling, virtual sensor, cross-correlation, self-recovery, fault-tolerant sensor, signal disturbance

## Abstract

A few prosthetic control systems in the scientific literature obtain pattern recognition algorithms adapted to changes that occur in the myoelectric signal over time and, frequently, such systems are not natural and intuitive. These are some of the several challenges for myoelectric prostheses for everyday use. The concept of the virtual sensor, which has as its fundamental objective to estimate unavailable measures based on other available measures, is being used in other fields of research. The virtual sensor technique applied to surface electromyography can help to minimize these problems, typically related to the degradation of the myoelectric signal that usually leads to a decrease in the classification accuracy of the movements characterized by computational intelligent systems. This paper presents a virtual sensor in a new extensive fault-tolerant classification system to maintain the classification accuracy after the occurrence of the following contaminants: ECG interference, electrode displacement, movement artifacts, power line interference, and saturation. The Time-Varying Autoregressive Moving Average (TVARMA) and Time-Varying Kalman filter (TVK) models are compared to define the most robust model for the virtual sensor. Results of movement classification were presented comparing the usual classification techniques with the method of the degraded signal replacement and classifier retraining. The experimental results were evaluated for these five noise types in 16 surface electromyography (sEMG) channel degradation case studies. The proposed system without using classifier retraining techniques recovered of mean classification accuracy was of 4% to 38% for electrode displacement, movement artifacts, and saturation noise. The best mean classification considering all signal contaminants and channel combinations evaluated was the classification using the retraining method, replacing the degraded channel by the virtual sensor TVARMA model. This method recovered the classification accuracy after the degradations, reaching an average of 5.7% below the classification of the clean signal, that is the signal without the contaminants or the original signal. Moreover, the proposed intelligent technique minimizes the impact of the motion classification caused by signal contamination related to degrading events over time. There are improvements in the virtual sensor model and in the algorithm optimization that need further development to provide an increase the clinical application of myoelectric prostheses but already presents robust results to enable research with virtual sensors on biological signs with stochastic behavior.

## 1. Introduction

Advances in the intuitive and natural prosthetic control with multiple degrees of freedom could significantly improve the quality of life of amputees [1,2,3]. However, a robotic hand based on non-invasive techniques is still a challenge in real application [4], especially when dealing with long-term usage. The myoelectric signal changes over time explain the difficulty of applying an intuitive and natural system. The myoelectric signals of a human being may change for reasons such as electrode conductivity changes that may be due to transpiration or environmental humidity, muscle fatigue, atrophy or hypertrophy, electrode displacement on the skin, changes in movement execution and the force intensity applied by the user [5,6,7].

The development of systems adaptive to these changes in the sEMG signal is necessary to obtain more intuitive and natural control myoelectric prostheses, but most of the recent developments are not adapted to such changes [4,5,6,7,8,9,10,11]. The sEMG signal is a stochastic time-series based on the physiological muscle properties and the subject muscle contraction form [12]. The same stochastic behaviour that provides the sEMG signal hinders its estimation.

Several solutions have been developed to reduce the interference in the acquired biomedical signals. However, a residual interference of these interferences still presents [13,14,15,16,17,18,19,20]. The signal contamination by motion artifacts causes data irregularities. Nonetheless, the effects of motion artifacts can be reduced by proper design of the electronic circuitry and set-up, but not eliminated [21]. The ECG interference is difficult to remove with conventional filters because the contamination overlaps with the sEMG signal in both the time domain and frequency domain [22]. For these reasons, it is essential to design classification systems that are robust enough to operate on signals containing such artifacts or can detect these artifacts so that signals containing them are discarded.

Virtual sensors are an emergent and intelligent tool which have been successfully used in other fields [23,24,25,26]. Usually, they are used to replace physical sensors [25,27,28]. They can also be used as part of fault detection methodologies, where their output is compared to the corresponding sensor [24,29,30,31,32]. The concept of virtual sensors is also present in studies in the context of wearable sensors [27,33] and physiological signals [34]. In Ref. [33], a multi-layer task model based on Hidden Markov Model (HMM) was presented and applied in the context of gait analysis. This virtual sensor approach research confirms the application effectiveness while maintaining high efficiency and accuracy. The virtual sensing service presented in [35] was used to estimate human body temperatures of various parts of the body by integrating human physiological models with measurable sensor data. In [34], a virtual sensor estimated the respiratory rate performance for time intervals, starting from a single-lead electrocardiogram signal.

The operation principle of these sensors is based on the mathematical model estimation of the collected data. The novel approach of the extensive fault-tolerant motion classification system consists of the use of the virtual sensor concept to reduce the impact over time of the sEMG signal degradation, combined with a fault-tolerant signal quality analysis detector. This study evaluated the five most common contaminants in sEMG signals [7,11,36]: motion artifacts, amplifier saturation, electrode displacements, power line interference and ECG interference. The study in [11] of signal contamination insertion and detection presents a one-class Support Vector Machine (SVM) successfully employed to detect a variety of contamination in sEMG signals with different SNR levels. There are also studies [11,36] that specifically identify which contaminant is present in the signal. Moreover, a signal quality analysis system in conjunction with re-training of the classifier in the removal of the contaminated channel is presented in [7].

The purpose of a virtual sensor model is to produce a signal output model independent of the physical acquisition of the signal of interest. The surface electromyography signal modeling is designed combining concepts of multichannel and their cross-correlation to replace degraded signal channels. This approach is referred to as multichannel cross-correlation. The objective of this new processing system is to maintain the classification accuracy after some signal degradation without the need for any retraining or calibration.

In this research, two types of sEMG signal modeling are evaluated: Time-Varying Autoregressive Moving Average (TVARMA) and the Time-Varying Kalman filter (TVK). The TVARMA models have already been used to improve non-stationary signal models [37]. Furthermore, the Kalman filter model has been used extensively in other fields [37,38,39,40,41,42], where it is considered an extremely efficient and flexible signal processing tool, and it is also employed in other virtual sensor enforcement [31].

Moreover, the system was evaluated in the characterization of seventeen hand-arm segment movements and the resting positions through the sEMG signals of twelve surface electrodes positioned on the upper limb. Regarding the number of subjects, ten non-amputee subjects and ten amputee subjects from the Non-Invasive Adaptive Hand Prosthetics (NINAPro) database were used to evaluate the mean classification accuracy of the system presented in this work.

## 2. Methods

### 2.1. System Overview

The processing and classification of the sEMG signals can be divided into the following seven experimental procedures to simplify the designed method: database loading, contaminated signal simulation, pre-processing, fault-tolerant detection, virtual sensor, feature extraction, and classification. The experimental system starts with the loading of NINAPro sEMG database. Before the common step where the data is preprocessed (filtering, rectification, and normalization), the stage of simulated contamination signals is composed of sixteen cases of contaminated electrode studies for each one of five types of contamination simulated from real examples of signal contamination. 

The researchers in [43,44,45] reported differences in the classification accuracy with the processing with pre-recorded data and real-time performance. Signal processing with pre-recorded data typically presents a higher movement accuracy. Consequently, the fault-tolerant detection, virtual sensor production, and feature extraction were performed online by scanning each of twelve-channel sliding windows to providing a suitable solution to the replicate real-life situation. However, the classification step was performed offline to perform the statistical analysis of the results. The features extracted of each case study are saved separately for further classification.

The sensor fault-tolerant detector (SFTD) performs quality analysis for sEMG signals using a two-class SVM, where the training occurs with selected signals. The training occurs in advance with sEMG signals with real acquired noise, several samples of clean sEMG signals, and their contamination based on motion artifacts, amplifier saturation, electrode displacements, power line interference and ECG interference. Only a few samples from the database of both intact and amputated subjects were used for the SFTD training. The objective was to train the detection sensor for the different amplitude variations of the sEMG signal. The same trained SFTD is then used for all subjects analyzed. When the SFTD detects that there is contamination in more than 70% of the signal for the last 3 s analyzed, the virtual sensor performs the signal modeling of interest for TVARMA and TVK models. One limitation of the response of the SFTD is that virtual sensor activation does not occur for noise bursts lasting less than 2.1 s.

**Figure 1:**
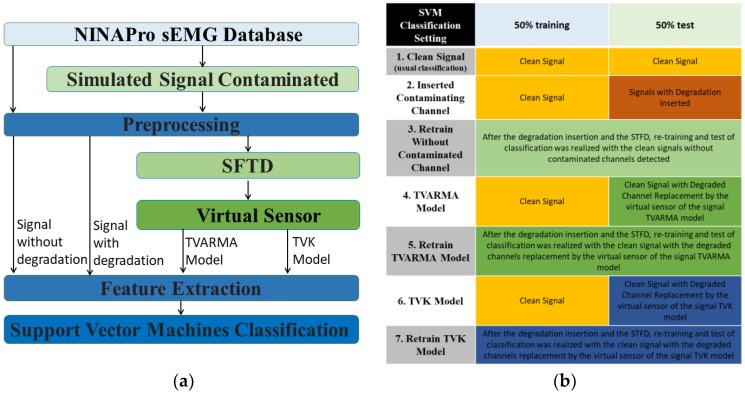
The experimental procedures (**a**) Flowchart of the method performed; (**b**) Description of SVM classification setting.

The features were selected based on results obtained in other studies [46,47]. The features Mean Absolute Value (MAV), Root Mean Square (RMS), Wave Length (WL), Maximum Fractal Length (MFL) and Power (PWR) are extracted. Each procedure is detailed in the next subsections. The experimental procedures can be seen in Figure 1a. All data saved from each subject have the same number of samples. The features extracted were used in the multi-class non-linear SVM classification in different analyses of setting classification cases, according to Figure 1b. The first analysis case corresponds to the usual classification, where 50% of the signal data without degradation were used for training and the other 50% for the test. The signal without degradation or the original signal was referred in this study as the clean signal. For cases 2, 4 and 6, the classifier training still remains using 50% of the clean signal. However, the other 50% of the corresponding data of the test are replaced with the signals with degradation inserted (case 2), the virtual sensor signal with the TVARMA model (case 4) and the TVK model (case 6). Case 3 analyzes the retraining of the classifier and test without the degraded channel detected by the SFTD. The last cases analyze the re-training of the classifier and test with the clean signal dataset with the replacement of the degraded windows by the virtual sensor with the TVARMA model (case 5) and the TVK model (case 7). In these two last cases, the training and the test were performed with the replacement of the signal by the models at all points detected by the SFTD.

### 2.2. NINAPro sEMG Database

**Figure 2:**
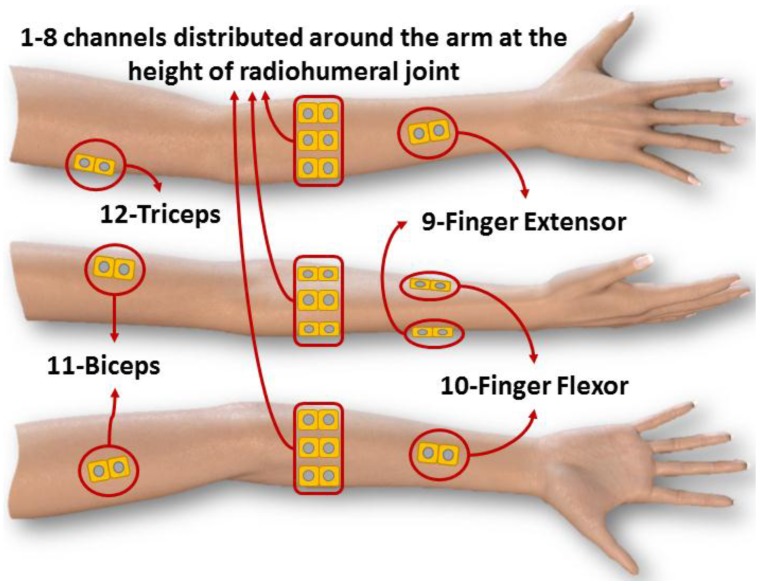
The positions of the electrodes.

The NINAPro project provides to the scientific community a database of sEMG signals. This database utilizes 12 active wireless electrodes of the DelsysTM TrignoWireless System^®^ [48]. The twelve electrodes are placed on the forearm, with eight uniformly spaced electrodes just beneath the elbow at a fixed distance from the radiohumeral joint, two on the flexor digitorum and the extensor digitorum, and two electrodes on the main activity spots of the biceps and the triceps. Figure 2 demonstrated the positions of the electrodes on the arm. NINAPro data is acquired using a NI-DAQ PCMCIA 6024E platform (National Instruments, Austin, TX, USA) at a rate of 2 kHz, 12 bits and with a lower than 750 nV RMS [49].

The timestamp was based on the virtual model of the orientation screen after the realignment treatment of each movement’s limits and the start and end time adjustment [50]. The timestamp generated from the stimulus videos was used to segment the signal. This study also does not monitor the subject-applied force and does not use any feedback procedure.

**Figure 3:**
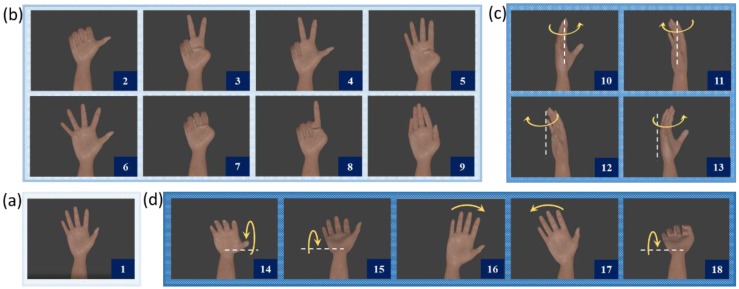
The hand-arm segment movements: (**a**) rest position; (**b**) hand movements; (**c**) Rotational movements; and (**d**) Wrist movements. The sequence of movements from 1 to 18: rest position; thumb up; flexion of ring and little finger, thumb flexed over middle and little finger; flexion of ring and little finger; thumb opposing base of little finger; abduction of the fingers; fingers flexed together; pointing index; fingers closed together; wrist supination and pronation (rotation axis through the middle finger); wrist supination and pronation (rotation axis through the little finger); wrist flexion and extension; wrist radial and ulnar deviation and wrist extension with closed hand.

During a signal acquisition session, a subject was required to perform six repetitions of 17 distinct movements (i.e., a total of 102 movements) which were interspersed with periods of rest in which the subject’s hand was in the resting position. The movement is performed for 5 s, interspersed with pauses of 3 s to allow the volunteer to rest. The rest position and the 17 distinct movements are presented in Figure 3. 

The first ten non-amputee subjects (aged 29 to 45 years) and ten amputee subjects (aged 32 to 67 years) of the NINAPro database were evaluated in this study. One amputee volunteer of NinaPRO database had an extremely noisy signal and was consequently excluded from the analysis. In these subjects, the SFTD detected similarities among the types of degradation evaluated in almost all the channel windows. The number of volunteers kept the same the number of amputee subjects and non-amputee subjects. The clinical characteristics of the analyzed amputated subjects provided by NINAPro are listed in Table 1.

### 2.3. Preprocessing

The pre-processing step aims to perform the digitized signal segmentation, rectification, and normalization. In real-time applications of prostheses, the classification is frequently based on the features extracted from the segmentation by sliding windows [51]. In this type of segmentation, the analysis window slides along in increments, adding new collected data and discarding the oldest data [52]. Majority voting strategies are commonly used to minimize the classifier output error when dealing with sliding windows [51,52]. Other studies suggest that the controller delay has to be as approximately 100 ms [29] and the top limit must be of roughly 300 ms [8,48,53].

The study in [48] analyzed the mean classification accuracy according to the window length variation in the NINAPro database. The study used sliding windows of 100, 200, and 400 ms with an increment of 10 ms for all analysis windows and they obtained the best accuracy with a window length of 400 ms. However, they did not analyze whether the effects of changing window length and increment variation were significant regarding the subject and the movement change.

Some researchers have already demonstrated that classification accuracy increases when the pattern recognition is performed on larger data windows [47,50,52,53]. However, this ends up increasing the time which is required to collect and process a more extensive dataset [52]. Thereby, a more significant amount of data results in features with lower statistical variance, which increases classification accuracy. The optimal mean classification accuracy for the different sliding windows is dependent on both the classifier and the feature extracted. Therefore, comparing results between studies with different characteristics are difficult. Thereby, this study chooses to use the sliding window size of 300 ms with the increment of 75 ms most common in the literature.

The Butterworth filter used on the signals was a digital band-pass filter of order 20 with a frequency range of 20 to 500 Hz. The sampling frequency was 2 kS/s. The normalization was performed separately for each channel considering all channel data. This method is not suitable for online processing. The online normalization must be standardized by a calibration procedure capturing the muscle signals in rest time and a moment of maximum voluntary contraction (MVC). However, the NINAPro database does not contain this information for calibration [54,55,56].

### 2.4. Sensor Fault-Tolerant Detector (STFD)

**Figure 4:**
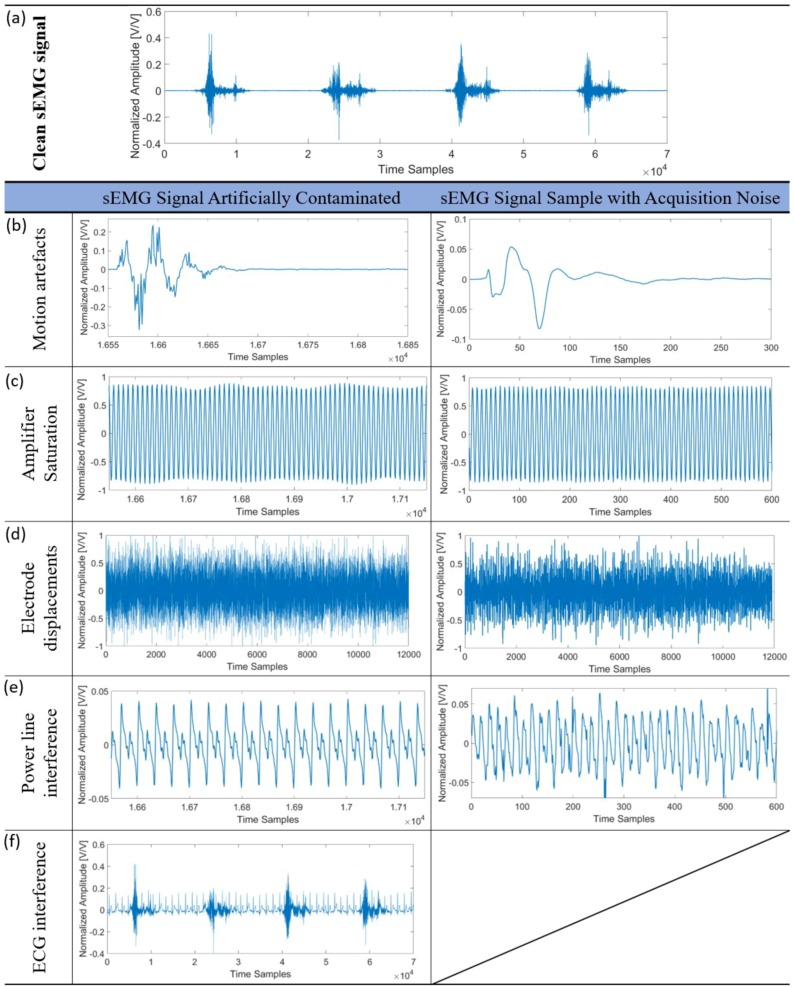
The comparison of each contaminant insertion in the clean sEMG signal of the subject 1 sample in four repetitions of movement 7. The clean sEMG signal sample in (**a**) is artificially contaminated by Motion artefacts in the first column in (**b**), by Amplifier Saturation in the first column in (**c**), by Electrode displacements in the first column in (**d**), by Power line interference in the first column in (**e**) and by ECG interference in the first column in (**f**). The second column in (**b**), (**c**), (**d**) and (**e**) are the sEMG signal samples with the acquisition of real noise.

The SFTD performs a quality analysis of sEMG signals based on the presence or absence of contaminants in the sEMG signal. The SFTD uses a two-class SVM that classifies a signal with or without contamination. The disturbances were simulated by the Matlab^®^ R2016b software using real signal contamination data samples as standard. In Figure 4 a comparison between each contaminant insertion in a clean sEMG signal sample with examples of a sEMG signal sample with acquisition noise can be observed. Each artificial contamination was approximated to a real acquisition of contaminated signal, except for ECG interference. The sEMG signals with actual acquisition of ECG interference are not possible in the forearm.

The correct detection of the occurrence of disturbances in the sEMG signal can allow the application of techniques to reduce the impact on movement classification accuracy. The contamination detection by the SFTD in sEMG signals was based on the results described in [11]. Their research already used the SVM trained only with clean signals and tested with artificially contaminated signals with different SNR levels. Their results show that a one-class SVM could be successfully employed to detect a variety of contaminants in sEMG signals. Differently, this proposed study sought to deepen the SFTD knowledge with the signals training with contamination and observed in real acquisitions of sEMG signals without varying the SNR level.

The data used for the SFTD training are composed of samples from some channels of the NINAPro database and real samples of contamination. The training occurred in advance of all signal processing. The signal quality test occurs for each sliding window of the 12 electrode channels. The developed method does not compromise online processing and can be applied to any subject. The operation analysis of this fault-tolerant detection requires a broad system for study the identification accuracy of the 5 types of contaminants. Therefore, it is essential to specify the simulation of the contaminated signal.

### 2.5. Signal Contamination Simulation

Some studies identify which contaminants are present in the signal [36]. However, this information is not relevant if the focus is to find a unique solution for all contamination cases. The classifier retraining disregarding the contaminated channels obtained significant results to maintain classification accuracy. Nevertheless, other studies observed that there is a processing cost to update the classifier only with the channels without contamination [7,57]. Also, there is not yet a thorough study in the processing cost to retrain the classifier after the end of the contaminant occurrence.

The performance analysis of this new approach which includes the virtual sensor in conjunction with STDF was carried out through different study cases for each contaminant type. Sixteen cases of contamination were examined for each contaminant type, where, in each case, artifacts were inserted into one channel or a combination of channels. The channels which were contaminated in each case are described in Table 2. The research in [58] related the impact of channels combination in the mean classification accuracy of the NINAPro database, demonstrating that include the signals of the biceps and triceps are a positive influence on the movement recognition. The channel combinations analyzed was select for the influence verifies of each channel and the influence of acquisition region of the upper limb. It essential note that this study not evaluated the variation effect of SNR and not changing the gain of the artifact signal before insertion.

The simulated contamination covers the entire channel period for each channel degradation case study before window segmentation. The motion artifact signals simulated to contaminate sEMG signal were estimated models based on the real signals acquired according to the tests described in other studies [7,36]. The electrode displacement artificially contamination was generated by Added White Gaussian Noise (AWGN) of 15 dB [36]. The amplifier saturation was implemented by addition of six sine waves with a random frequency between 200 and 240 Hz as analyzed in the sEMG signal sample with acquisition amplifier saturation noise and based on another study [11].

A sine wave, which has a frequency of 60 Hz, its harmonics, and an amplitude of 0.4 V, was added to the signals to simulate power line interference. The ECG artificial interference occurred with ECG database available from PhysioBank ATM of Physionet (http://www.physionet.org) with the same sampling frequency of sEMG signal. The ECG interference does not occur in the electrode position in this work that is why the acquisition of real noise is not demonstrated. However, the interference detection was analyzed for the application of myoelectric protheses and the ECG interference is present in severe cases of left upper limb amputation, depending on where the electrodes are positioned and whether Targeted Muscular Reinnervation (TMR) occurred. The ECG signal was normalized and added to sEMG signal with maximum amplitude was established at 0.2 for the detection tests.

### 2.6. Virtual Sensor

The initial idea of this study was conceived through studies of cross-correlation coefficient analysis applications involving investigation of the crosstalk among different sEMG channels [59] and analysis of the degree of synchronization between the surface electromyography recordings for the two muscles [60]. We approached the idea that there is some cross-correlation coefficient between the muscular fibers or muscles of the same hand-arm segment in the performance of the movement and this idea was called multichannel cross-correlation (MCC).

The cross-correlation ($r_{xy}$) between two sEMG signal channels (x,y) was normalised ($c_{xy}$) for the number of channels (M), which is the same for all channels. The novel approach called multichannel cross-correlation for utilization on the virtual sensor provides the base of the signal modelling of TVARMA and TVK models. The MCC is a matrix which can be obtained through the percentage contribution ($p_{xy}$) of each cross-correlation coefficient between the M acquired channels with Equation (1):

(1) $$p_{xy}=\{\begin{array}{c} \frac{c_{xy}}{\sum_{i=1}^{M}c_{xy,i}},\;x\neq y\\ 0,\;x=y \end{array}$$

For 12 sEMG signal channels, *x* and *y* vary with the interval (1,12) providing a multichannel cross-correlation matrix of order 12 × 12 as can be seen in (2):

(2) $$p_{xy}=\left[\begin{array}{cc} \begin{array}{c} \begin{array}{cc} 0 & p_{2,1}\\ p_{1,2} & 0\\ p_{1,3} & p_{2,3} \end{array}\\ \begin{array}{cc} \vdots & \vdots \\ p_{1,12} & p_{2,12} \end{array} \end{array} & \begin{array}{c} \begin{array}{ccc} p_{3,1} & \cdots & p_{12,1}\\ p_{3,2} & \cdots & p_{12,2}\\ 0 & \cdots & p_{12,3} \end{array}\\ \begin{array}{ccc} \vdots & \ddots & \vdots \\ p_{3,12} & \cdots & 0 \end{array} \end{array} \end{array}\right]$$

**Figure 5:**
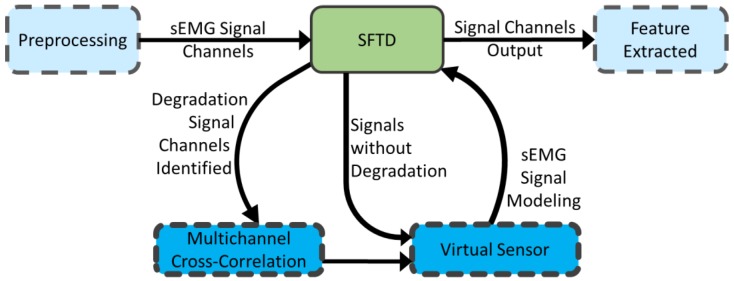
The operating logic of the SFTD with the virtual sensor.

The process of signal quality analysis and the signal modeling by the virtual sensor occurs for each examined window. Virtual sensor activation is performed by the SFTD. The SFTD adjusts the MCC matrix, removing contaminated channels when detected contaminated signals above 70% of the windows in the last 3 s. After, the SFTD transmit the matrix and the signals for the virtual sensor. The correlation coefficient between sensor responses has already been used differently as the input of one virtual sensor [61]. The virtual sensor replaces sEMG contaminated signal channel based on the SFTD analysis to the sEMG signal modeling using the TVARMA and TVK models. This operating logic can be seen in Figure 5.

#### 2.6.1. TVARMA Model

The proposed sEMG signal TVARMA model for the virtual sensor at time n can be represented in (3) and was based on the sEMG model proposed in [37,62]. Each sEMG signal channel segment of the virtual sensor $y_{channel}\left(n\right)$ is established as a linear combination of the previous output samples, plus the previous input samples $u_{channel}\left(n\right)$ and an error term of the noise error present $e_{channel}\left(n\right)$, which is independent of past samples:

(3) $$y_{channel}\left(n\right)=\sum_{i=1}^{P}a\left(i,n\right).y_{channel}\left(n-i\right)+\sum_{j=1}^{Q}b\left(j,n\right).u_{channel}\left(n-j\right)+e_{channel}\left(n\right)$$

where the a(i,n) is the time-varying autoregressive (AR) coefficient, b(j,n) is the time-varying moving average (MA) coefficient and the indexes P and Q are the highest orders of the AR and MA models, respectively. The model has the model orders set to 4 for P and 2 for Q.

The input samples $u_{channel}\left(n-j\right)$ can be obtained through Equation (4), which uses the respective channel of interest’s column of the MCC matrix and the other channel samples considered as clean signals from sEMG $\vec{y}\left(n-i\right)$. The channels with known contamination have the correlation coefficient line set to zero:

(4) $$u_{channel}\left(n-j\right)={\left({\vec{p}}_{x,channel}\right)}^{T}.\vec{y}\left(n-i\right)$$

where ${\vec{p}}_{x,channel}$ is the respective values column of the percentage contribution of the cross-correlation coefficient between the channel of interest and the other channels.

A finite-order base function f(n,m) was imposed for the functions of parameters a(i,n) and b(j,n) to obtain a better model in Equations (5) and (6). These functions were proposed in other studies of TVARMA models [62,63]:

(5) $$a\left(i,n\right)=\sum_{m=0}^{V}\alpha \left(i,m\right)f\left(n,m\right)$$

(6) $$b\left(j,n\right)=\sum_{m=0}^{V}\beta \left(j,m\right)f\left(n,m\right)$$

where f(n,m) with m=0, 1, 2, …, V and n=0, 1, 2, …, N−1 is the base function that needs to be selected. The parameter functions α(i,m) and β(j,m) represent the expansion of the parameters with V, which is the maximum number of base sequences [62]. Therefore, the proposed sEMG signals TVARMA model is finally defined as shown in (7):

(7) $$y_{channel}\left(n\right)=\sum_{i=1}^{P}\sum_{m=0}^{V}\alpha \left(i,m\right).y_{channel}\left(n-i\right)+\sum_{j=1}^{Q}\sum_{m=0}^{V}\beta \left(j,m\right).u_{channel}\left(n-j\right)+e_{channel}\left(n\right)$$

#### 2.6.2. TVK Model

The TVK model of sEMG signals for the virtual sensor is based on Kalman state estimator that is given a state-space model with satisfactory known inputs $u_{channel}\left(n\right)$, white process noise w(n), and white measurement noise v(n). The virtual sensor measurement $y_{channel}\left(n\right)$ and the state equation x(n) can be represented by Equation (8):

(8) $$\begin{array}{l} x\left(n+1\right)=Ax\left(n\right)+Bu_{channel}\left(n\right)+Gw\left(n\right)\\ y_{channel}\left(n\right)=Cx\left(n\right)+v\left(n\right) \end{array}$$

where A, B, C, and G are state matrices.

The $y_{channel}\left(n\right)$ and $u_{channel}\left(n\right)$ are estimated in the same way as reported for the TVARMA models. The estimator generates output estimates $\hat{y}\left[n|n\right]$ and state estimates $\hat{x}\left[n|n\right]$ using all available measurements up to $y_{channel}\left(n\right)$. The state estimator can be represented as in (9):

(9) $$\hat{x}\left[n+1|n\right]=A\hat{x}\left[n|\left(n-1\right)\right]+Bu_{channel}\left(n\right)+M\left(y_{channel}\left(n\right)-C\hat{x}\left[n|\left(n-1\right)\right]\right)$$

where M is the innovation gain, which updates the prediction $\hat{x}\left[n+1|n\right]$ using the new measurement $y_{channel}\left(n\right)$, and it is determined by solving an algebraic Riccati equation in (10), where P solves the corresponding equation:

(10) $$M=PC^{T}{\left(CPC^{T}+R\right)}^{-1}$$

The final estimator $\hat{y}\left[n|n\right]$ for the virtual sensor has the following output equation:

(11) $$\left[\begin{array}{c} \hat{y}\left[n|n\right]\\ \hat{x}\left[n|n\right] \end{array}\right]=\left[\begin{array}{c} C\left(I-MC\right)\\ I-MC \end{array}\right]\hat{x}\left[n|n-1\right]+\left[\begin{array}{c} CM\\ M \end{array}\right]y_{channel}\left[n|n\right]$$

The matrices of the TVK model were set with the identity matrix. The sEMG signals models were applied to all sliding windows detected by the SFTD for each subject in the sixteen different cases reported for each simulated signal contamination.

### 2.7. Feature Extraction

The efficiency of the sEMG signal classification system depends on the choice of features [64]. However, the features selected may be redundant or even irrelevant [65]. Each feature is not equally necessary for a specific task. The features were selected based on the result of the classification of other studies [46,53,66] with the same database and the evaluation of characteristics for sEMG [47].

The characteristics were extracted from each sliding window and the features selection in the time domain is directly related to the MMC concept, which analyses the percentage contribution of each cross-correlation coefficient of two sEMG signal channels in the time domain. It is important to emphasize that no feature reduction technique was used. The combination of the MAV, RMS, WL, MFL, and PWR features presented better mean classification accuracy results for the analyzed subjects. 

### 2.8. Classification with Support Vector Machines

The non-linear SVM classification with radial basis function (RBF) kernel was implemented in the sEMG signals classification of all simulated cases. The kernel functions parameters were selected by a search algorithm of the best result for each subject. The multi-class definition technique for classifying movement was eighteen binary classifications of one versus all. In more than one positive class case, the algorithm selects the class that is farthest away from the hyperplane that separates each binary classification.

The majority voting technique is used as a post-processing mechanism considering last three window classification. Also, the k-fold complimentary technique was applied for improving the reliable accuracy test with the small number of samples in which the model is trained and tested. The tests are carried out in all possible different input conditions forming 20 k-folds for the six available movement repetitions. For each k-fold, three of the six repetitions (50% of the dataset) were selected for the training model and three (50% of the dataset) for testing.

The classification procedure was applied to seven different settings, according to the Figure 1b. The classification using the signal without contamination performed the usual training and test in the average of all possible k-folds. The classification analyses of the signal contamination were performed by training the classifier with the data part of the signal without degradation and the test with the other corresponding part in each separate case for the signal with degradation inserted. The classifications using the virtual sensor occur similar to the previous, the data part of the signal without degradation was used for training, but the signals with TVARMA model and TVK model replace the degradation inserted detected for STFD for the test.

The seven different settings include the re-training practice of the classifier for comparative performance analysis. The classification re-training without the degraded channel was performed when 80% of the windows were considered contaminated by the SFTD. The classification with re-training using the two signals modeled was made through the contaminated signal replacement by the virtual sensor in the training and testing. For every sliding window that featured 70% of contamination detected by the SFTD in the last 3 s, the virtual sensor replaced the sEMG signal by the modeled signal. This threshold was established based on another study [57], which introduced the idea of contaminant temporality and the return to reconsideration of the contaminated channel. Each subject was analyzed for different contamination types in different channel arrangements, and each analysis resulted in a specific confusion matrix of all k-folds each classification results.

### 2.9. Experimental Statistical Analysis

Design and analysis of three-factor experiments were entirely randomized and used for the statistical validation of the test methodology. A Design of Experiments full factorial design [67] was realized with the mean accuracy response variable and the controllable factors: the classification setting type, the contaminant signal inserted and the variation cases of channel contaminations. The model used follows in (12):

(12) y_ijk=μ+α_i+β_j+γ_k+(αβ)_ij+(αγ)_ik+(βγ)_jk+(αβγ)_ijk+ε_ijk

where y_ijk corresponds to the level i response in repetition j, μ corresponds to the general average, α_i corresponds to the effect of each level i and ε_ijk corresponds to the error of level i in repetition j.

Analyses of variance (ANOVA) and multiple comparisons also were used for providing a statistical test, which makes possible to assert whether the various group’s average differences are significant or not. Two averages are significantly different when their intervals are disjointed. When their intervals overlap, they are not significantly different.

## 3. Results

The signal processing was analyzed for ten intact subjects and ten amputees. It is important to note that the proposed method using a virtual sensor is independent of the pattern algorithm because the virtual sensor is applied before classification. The SFTD detection and the movement classification used the algorithm SVM. However, there is no dependency on this algorithm. This new extensive fault-tolerant system could use any other algorithm. The SVM is widely used in sEMG signals [46,68,69,70,71,72] and was selected to compare and evaluate the effectiveness of this new system.

### 3.1. SFTD Results

For the correct interpretation of the results, an analysis of the detection of the windows contaminated by the SFTD is necessary. The SFTD detection accuracy of 85.31 ± 24.88% and false-positive recognition for the channels that non-received the artificial contamination of 8.18 ± 17.52% demonstrated the sensibility of sensor detection. All acquired signal has noise, even after preprocessing, since there may be noises in the frequency range of the sEMG signals.

The SFTD has a differentiation in detection depending on the analyzed contaminant. The ECG and power line interference detection have obtained lower precisions. These contaminants present an effect of lower significance than the other three disturbances. The detection of the ECG and power line interferences in channel 12, which corresponds to the electrode positioned on the triceps, obtained a precision lower than 20% of the average of the other channels. The acquired channel 12 also features the low representativity of the sEMG signal compared to the noise level of the channel. This low representativity may explain the accuracy detection decrease of the SFTD since channel 12 does not seem to be significantly relevant to the movement classification.

### 3.2. Movement Classification Results

**Figure 6:**
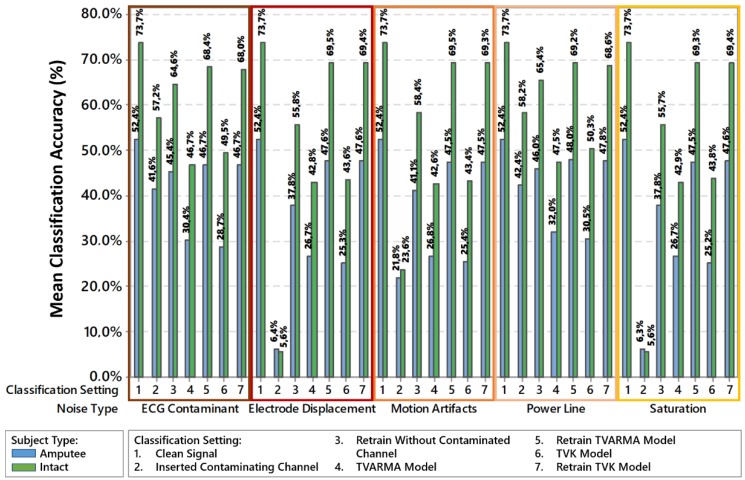
Classification setting comparison for each contaminant insertion type and the clean sEMG signal classification.

Figure 6 presents the mean classification accuracy for all 16 noise insertion cases with different channel combinations. 

In Figure 6, it is possible to realize that each contamination type impairs the classification differently in comparison to the clean signal classification (legend 1), in which some contaminations are more expressively than others, such as saturation and displacement of electrodes. For contamination by electrode displacement and saturation, the substitution of the contaminated signal by the signals modeled with TVARMA (legend 4) and TVK (legend 6) recovers at least 30% of the mean accuracy classification. For contamination by movement artifacts, recovery is at least 20% for intact subjects and approximately 4% for amputated subjects. For ECG contamination and power line interference, the contaminated signal replaced by the virtual sensor models only further damages the signal.

In the comparison of the classifier retraining with removal of the contaminated channel (legend 3), the classifier retraining with the contaminated channel entirely replaced by the virtual sensor using the TVARMA model (legend 5) and using the TVK model (legend 6) demonstrate the improvement or recovery of the mean accuracy classification results after the signal contamination. The classifier retraining method with removal of the contaminated channel recovers 50% of the mean accuracy in the intact subjects and 30% in the amputees for electrode displacement and saturation, 30% in the intact subjects and 20% in the amputees for motion artifacts, 7% in the intact subjects and 3% in the amputees for ECG and power line interference. The classifier retraining method of replacing the degraded channel by the signal modeled by the virtual sensor in the TVARMA and TVK models achieves a mean classification accuracy of at most 5.7% below the clean signal classification.

### 3.3. Experimental Statistical Results

The ANOVA obtained results that disregard the possible factors such as subjects and movements because they have already proven that they influence the mean classification accuracy significantly [46,73]. The ANOVA used a 95% confidence interval for all response factors. The significant F-ratio is used to investigate whether the difference among the sample means is significant or just matter of sampling fluctuations. The F-test on main effects and interactions follows directly from the expected mean squares. The calculated value of F-ratio above of the table value of P define if data is significant. The factors classification setting (F = 6992.86 > *p*-value = 0.0), the contaminant signals inserted (F = 194.02 > *p*-value = 0.0) and the cases of variation of channel contaminations (F = 1299.08 > *p*-value = 0.0) indicate that all the factors are individually significant, and their interactions are also significant for mean classification accuracy. That is, each method can achieve better results than another depending on the contaminant and the analyzed combination of noise insertion. 

## 4. Discussion

Analyzing the contaminants detection with the other two similar studies [11,36], SFTD demonstrated very similar performance to the study [11], which also uses SVM and has difficulty detecting the same contaminants with low SNR. It is more difficult to compare with the study [36], that identifies which noise is present in the signal among the contaminants. Since its classification accuracy is based on the correct identification of the noise present among the possible contaminants, it is impossible to compare it with the SFTD. However, the study [36] also uses an SVM algorithm and reinforces the potential of applying this method to identify and mitigate the influence of contamination on acquired sEMG signals. Thereby, after using the SFTD, the presented detection method obtained a suitable solution for identification of contaminants in the sEMG signal. However, the selection of additional features for better detection of ECG and power line interference is a possibility to improve the sensor detection.

**Figure 7:**
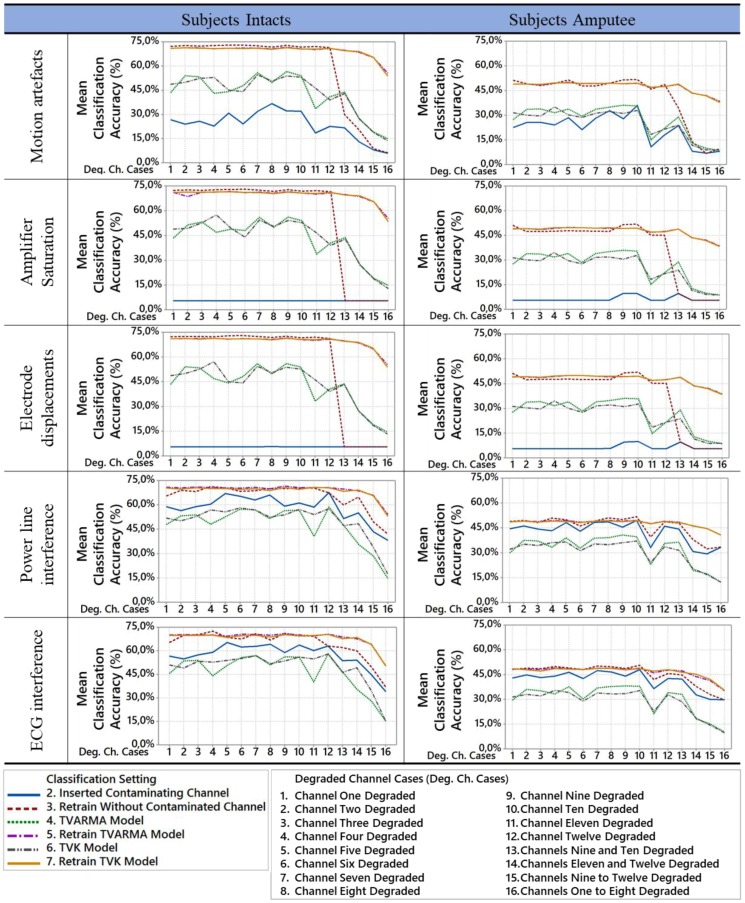
Degradation channel cases comparison for each classification setting.

The classification setting method analysis for the 16 cases of insertion of contamination in different combinations of channels can be visualized in Figure 7. Regarding the previous analysis, this figure shows that the classifier retraining method without the contaminated channel does not maintain its performance when more than one channel has any contaminant insertion. However, the classifier retraining method with replacement signal by virtual sensor models maintains up to 20% variation for the worst case of degradation (legend 16). The already widely used method of removing the contaminated channel [7,57] has a lower performance in the movements characterization when more than one channel was degraded to the method proposed in this paper with the classifier retraining.

It is important to emphasize that the mean accuracy of the classification settings 5 and 6 are practically overlapped in Figure 7. This overlap demonstrates that it is possible that the chosen model between TVARMA and TVK is not significant. However, the classifier retraining method replacing the signal degraded by the virtual sensor signal increased the mean accuracy in the analysis of all contaminated cases.

The implementation in [57] of a self-adaptive neural network of self-retraining discarding the channels which considered with the looseness or electrodes misplacement was used for the improvement of acquired sEMG signals application. However, there is no artificial contamination, only the contaminations obtained during signal acquisition. The sEMG signal had already intrinsic known contaminants, which were identified using threshold for detection. The presented new system in this work is more complex and evaluates several other interferences. Nonetheless, both demonstrate the possibility of using detection systems to improve the movement classification.

The study in [7] already has a contamination simulation approach like the one implanted in this study. However, this other study tested contact artifacts, loose contacts, and baseline noise with different SNR levels. Although the other study in [7] does not use the same database and they focus on reducing the re-training time of the classifier after their detection sensor module, it is possible to make some comparisons with this work. The number of false-positive occurrences is lower in [7], but this may be a feature of the database used. In a comparison among the noises used, it is possible to affirm that they obtained a less accurate detection for loose contacts.

For the loss of the average accuracy classification after the contamination of one to three channels, this other study had a lower effect of the contaminants in the movement classification for the amputated subjects and non-amputated subjects. The worst case of three contaminated channels presented in the other study has a decrease of classifier accuracy by up to 15%. The comparison the same retraining method discarding contaminated channels of this work with the other study in [7] shows a lower decrease in accuracy for the same noise with more than one contaminated channel for the other study. However, it also showed less influence of the contaminants on the accuracy of the classifier.

Therefore, it is possible to affirm that this new fault-tolerant classification system obtained a suitable solution with better results than the already presented studies. Also, the other studies did not evaluate so many contaminants and cases of channel contamination. This new classification system when using classifier retraining with the virtual sensor signal recovered the mean classification accuracy to the maximum of 5.7% below the clean signal classification, and the worst case with eight contaminated channels obtained a maximum decrease in the mean classification accuracy of 15%.

This statistical analysis can be observed previously, however, the proximity of the results to the retraining methods with the virtual sensor models attained very close results. Thus, a new ANOVA was needed using only the retraining classifier methods with the TVARMA and TVK models for the virtual sensor in the classification setting type factor. This ANOVA results obtained showed that individual mean results are significant (F = 0.62 > *p*-value = 0.43), but their interactions with the other factors are not (F(combination channels, classification setting) = 0.21 < *p*-value = 0.999), F(combination channels, noise type) = 0.51 < *p*-value = 0.999), F(classification setting, noise type) = 0.25 < *p*-value = 0.908)). In other words, one model always has better results than the other when it changes the contaminant type or the variation of cases of channel contaminations and both together.

## 5. Conclusions

The proposed method can maintain over 60% of the classification accuracy of the clean sEMG signal when contaminated with electrode displacement and saturation and 20% when contaminated with motion artifacts, without a retraining or calibration procedure. For classifier retraining methods, the replacement of the contaminated channel by the virtual sensor model demonstrates the best results for all types of contaminants.

The statistical experimental results determined that the variation of the classification setting, the contaminant signal inserted and the cases channel contaminations significantly affect the mean classification accuracy. The comparison and statistical analysis of the virtual sensor method with the TVARMA and TVK models demonstrate that the TVARMA model obtains more significant mean accuracy for the variation of the contaminant signal inserted, for the variation of cases channel contaminations and both together.

The SFTD accuracy was obtained using a training base of intact subjects and amputees. It is may be possible to improve this accuracy by dividing the intact subjects and amputees for training SFTD. However, it is necessary the controlled acquisition of contaminants in amputees, and calibration may be required, because of the uniqueness of each amputee’s muscular integrity.

The quality analysis and virtual sensor model run online at the same time for both TVARMA and TVK models. The processing time of the virtual sensors with the SFTD was not computed. The classification is performed offline later to generate the results of the statistical tests. The algorithm of the proposed system can be optimized for the user online application in the next steps, considering the results analyzed in this work. Classification accuracy cannot be determined in most surveys since different movements and subjects are evaluated. The use of a public database such as NINAPro enables the scientific community to evaluate the contribution of this innovation. It is important to note that proposed technic of adaptation can be a form of complementation with several other approaches such as user adaptation with online feedback [74].

The proposed system is a complementary technique to increase the clinical impact of the myoelectric prosthesis or other applications with the same stochastic behavior signals. For example, EEG for seizure detection, ECG for QRS detection or arrhythmia classification. However, the next step in this research will be the improvement of virtual sensor model adaptation for frequency features and consequently the classification results enhancement. In parallel, an adjustment of SFTD method should be developed for ECG and power line interference. 

## Figures and Tables

**Table 1:** Clinical characteristics of the amputated subjects.

Subject	Age	Handedness	Amputated Hand (s)	Amputation Cause	Remaining Forearm (%)	Year Since Amputation
1	32	Right	Right	Accident	50	13
2	35	Right	Left	Accident	70	6
3	50	Right	Right	Accident	30	5
4	34	Right	Right and Left	Accident	40	1
5	67	Left	Left	Accident	90	1
6	32	Right	Left	Accident	40	13
7	33	Right	Right	Accident	50	5
8	44	Right	Right	Accident	90	14
9	59	Right	Right	Accident	50	2
10	45	Right	Right	Cancer	90	5

**Table 2:** Channels contaminated in sixteen cases for each contaminant type.

Case	Channels Contaminated	Case (Cont.)	Channels Contaminated (Cont.)
1	The 1st electrode of eight uniformly spaced electrodes	9	Flexor digitorum electrode
2	The 2nd electrode of eight uniformly spaced electrodes	10	Extensor digitorum electrode
3	The 3rd electrode of eight uniformly spaced electrodes	11	Bicep electrode
4	The 4th electrode of eight uniformly spaced electrodes	12	Tricep electrode
5	The 5th electrode of eight uniformly spaced electrodes	13	Flexor digitorum and extensor digitorum electrodes
6	The 6th electrode of eight uniformly spaced electrodes	14	Bicep and tricep electrodes
7	The 7th electrode of eight uniformly spaced electrodes	15	Flexor digitorum, extensor digitorum, bicep and tricep electrodes
8	The 8th electrode of eight uniformly spaced electrodes	16	All eight uniformly spaced electrodes
